# Metals and metallothionein evolution in snails: a contribution to the concept of metal-specific functionality from an animal model group

**DOI:** 10.1007/s10534-024-00584-3

**Published:** 2024-02-28

**Authors:** Reinhard Dallinger

**Affiliations:** Universität Innsbruck, Innsbruck, Austria

**Keywords:** Mollusca, Gastropoda, Metallothionein, Cadmium, Copper, Zinc

## Abstract

This is a critical review of what we know so far about the evolution of metallothioneins (MTs) in Gastropoda (snails, whelks, limpets and slugs), an important class of molluscs with over 90,000 known species. Particular attention will be paid to the evolution of snail MTs in relation to the role of some metallic trace elements (cadmium, zinc and copper) and their interaction with MTs, also compared to MTs from other animal phyla. The article also highlights the important distinction, yet close relationship, between the structural and metal-selective binding properties of gastropod MTs and their physiological functionality in the living organism. It appears that in the course of the evolution of Gastropoda, the trace metal cadmium (Cd) must have played an essential role in the development of Cd-selective MT variants. It is shown how the structures and Cd-selective binding properties in the basal gastropod clades have evolved by testing and optimizing different combinations of ancestral and novel MT domains, and how some of these domains have become established in modern and recent gastropod clades. In this context, the question of how adaptation to new habitats and lifestyles has affected the original MT traits in different gastropod lineages will also be addressed. The 3D structures and their metal binding preferences will be highlighted exemplarily in MTs of modern littorinid and helicid snails. Finally, the importance of the different metal requirements and pathways in snail tissues and cells for the shaping and functionality of the respective MT isoforms will be shown.

## Introduction

Based on findings in the animal group of Gastropoda (snails), the following review is dedicated to the evolutionary and functional intertwining of metallothioneins (MTs) with the three important d-block metals zinc (Zn), copper (Cu) and cadmium (Cd).

MTs are ubiquitous in eukaryotic organisms and have also been discovered in many prokaryotes (Blindauer and Leszczyszyn 2010). They are defined as predominantly low molecular weight proteins with a high proportion of cysteine (Cys) amino acid residues, via whose Sulphur atoms d-block metal ions are bound with high affinity. For a more detailed definition and description of these metal-binding proteins, one may refer to articles and reviews in the literature (Kägi and Schäffer 1988; Vašák 2005; Capdevila and Atrian 2011; Isani and Carpenè 2014; Ziller and Fraissinet-Tachet 2018; Krȩżel and Maret 2021). Among the many d-block metal ions that can bind to MTs in vitro, Zn^2+^, Cu^+^ and Cd^2+^ are the ones that most frequently form metal complexes with MTs also under natural conditions (Vallee 1979). Of these three metals, Zn and Cu are essential trace elements, while no essential function has yet been demonstrated for Cd, with the exception of some marine diatoms that use Cd as an essential component of their enzyme carbonic anhydrase (Lane et al. 2005). A major biological challenge is the remarkable similarity of Cd and Zn: both metals belong to the same group of elements, sharing their atomic structure and similar chemical and geochemical features (Vallee 1979). Therefore, most natural Zn ore deposits also contain small amounts of Cd, with an average concentration in the earth’s crust of 0.5 to 1 mg/kg, which is only about one hundredth of the corresponding Zn concentration (Sposito 1986). The similarity and common natural occurrence of the two metals is reflected in their biological behavior: Cd as a non-essential toxic trace metal can interfere with essential pathways and functions of Zn and displace the metal from functional molecules in biological systems (Brzóska and Moniuszko-Jakoniuk 2001; Tang et al. 2014).

It may therefore come as no surprise that the first description of an MT was related to the discovery of surprisingly high concentrations of Cd in the kidney of the horse (*Equus caballus*), where the metal was found to be apparently bound to this protein, along with much lower amounts of Zn (Margoshes and Vallee 1957; Kägi and Vallee 1960). A function of the newly discovered MT protein in connection with Cd^2+^, perhaps for its detoxification, therefore seemed to be a possible hypothesis to explain the high Cd content of this protein. Notably, however, only a few years after the first description of the Cd-containing MT, an MT isoform was isolated from the liver of the horse, which—contrary to the findings in the kidney—bound predominantly Zn and only little Cd (Kägi et al. 1974). Since then, many other MTs have been described containing mainly Zn and Cu, but only traces, if any, of Cd (Bakka and Webb 1981; Henry et al. 1994; Andrews et al. 1996), so that the opinion gained ground among well-known authors that the function of MTs can only be understood in the context of zinc metabolism (Vallee 1979, 1995; Brady 1982; Davis and Cousins 2000; Cousins et al. 2006; Krȩżel and Maret 2021). In fact, a direct involvement of the brain-specific isoform ZnMT-3 in protection against amyloid-ß toxicity through metal exchange between ZnMT-3 and amyloid-ß-Cu clearly proved a direct involvement of a Zn-specific MT in the pathology of Altzheimer's disease (Meloni et al. 2008). On the other hand, Klaassen and his colleagues were convinced that Cd exposure experiments with transgenic MT null mice provided evidence that the main function of MT in mammals is to protect the organism from the harmful nephrotoxic effects of Cd (Klaassen et al. 2003). The existence of functional Cu-specific MT variants and isoforms additionally illustrates the immense versatility of the members of the MT family (Calvo et al. 2017). At the molecular level, signaling pathways via the metal-responsive transcription factor-1 (MTF-1) and some other transcription factors, indicate that MT gene upregulation may depend, in a cell and isoform-specific manner, on the presence of elevated amounts of Zn^2+^, Cd^2+^ and Cu^2+^, and in many cases on a number of additional substances and stressors (Haq et al. 2003). Such seemingly contradictory findings and many different conclusions, along with false expectations, may have contributed to the fact that MTs have periodically been surrounded by an aura of mystery, elusiveness and ambiguity about their supposed “true” function (Palmiter 1998; Coyle et al. 2002).

Some important clarifications need to be made at this point. First, much of the initial progress in MT research was focused on vertebrate MTs (Vašák and Hasler 2000; Vašák 2005), so that our view of the structure and function of MTs is biased in favor of this relatively small group within the much more diverse animal kingdom. This has been rightly pointed out in some previous and more recent publications (Dallinger 1996; Isani and Carpenè 2014; Ziller and Fraissinet-Tachet 2018; Abdin et al. 2021). Secondly, the assignment of specific functions to MTs depends on how narrowly or how broadly one defines the concept of functionality in the biological context for proteins that complex metals with unprecedentedly high affinity and simultaneously exhibit a high degree of dynamic metal exchange capacity (Freisinger and Vašák 2013; Abdin et al. 2021).

In this review, I will try to approach the concept of MT functionality in the context of their metal binding properties through an evolutionary biology approach. For a holistic account, I also refer here to a series of papers that were published in recent years in collaboration with an international research consortium dedicated to understanding the evolution of snail MT functions in a functional context. This cooperative effort included researchers from different fields (biology, bioinorganic chemistry, structural biology, biochemistry and molecular biology) and was made possible by a number of research projects granted by the Austrian Science Fund (FWF).

Numerous authors have so far attempted to clarify the origin of MTs using methods of evolutionary biology. This usually involves the construction of phylogenetic trees with the aim of tracing MTs in different organismal kingdoms and animal groups back to an ancestral precursor form and elucidating their evolutionary-biological diversification, also with the aim of understanding functional relationships between the different MT lineages and genes. A phylogenetic classification of all known MT families was first provided by Binz and Kägi (1999). Many other authors have mainly explored phylogenetic relationships of MT subfamilies in different animals groups (Nemer et al. 1985; Lange et al. 1990; Stephan et al. 1994; Andrews et al. 1996; Valls et al. 2001; Capasso et al. 2003; Wan et al. 2005; Santovito et al. 2007; Janssens et al. 2009; Trinchella et al. 2008; Ragusa et al. 2009; Capdevila and Atrian 2011; Serén et al. 2014; Jenny et al. 2016; Nam and Kim 2017; Luo et al. 2020; Calatayud et al. 2021a). The approach to studying MT evolution in gastropods that I refer to here is very broad. This means that taxon-specific evolutionary alterations in the primary structure of MTs was and still is combined with studies of their metal-binding properties, their structure, and other biochemical and physiological traits. This makes it possible to track evolutionary innovations or metal-specific loss of function along and across different gastropod lineages, to infer them from each other and thus to understand them better. This has become possible because gastropods are, thanks to their multitude and diversity, an ideal animal group for such studies (Dallinger et al. 2020).

## A preliminary definition of some important terms

Before entering *medias res*, it may be helpful to define some important terms used in this article, also to avoid misunderstandings about their divergent use in other MT publications. These terms refer either to the metal-binding properties of snail MTs and MT isoforms or to their derived physiological functions in the snail or in other organisms under in vivo conditions.

In the case of metallothioneins, the term “metal binding affinity” refers to the question of how strongly a metal is bound by the corresponding protein’s thiolate ligands, either at individual binding sites or as the integral value of all binding sites together, typically expressed as metal binding constant (log_10_K_N_) (Scheller et al. 2017). Its absolute value for the different MT metal species is difficult to determine, depending on various physical–chemical boundary conditions such as the pH, the metal loading dynamics and cooperativity, redox potential, or temperature (Scheller et al. 2017, 2018). In general, the metal binding affinity in the various metal thiolate complexes follows a certain predetermined succession (Irving and Williams 1948), with an apparent stability constant for MT metal complexes in the following ascending order: Zn < Pb < Cd < Cu(I) (Kägi and Schäffer 1988).

The term “metal selectivity”, also referred to as “metal binding preference”, and the term “non-selectivity”, relate to the propensity of an MT or an MT subunit (domain) to bind and coordinate certain metal ions preferentially due to the peptide’s innate structural configuration. In most cases, these properties are investigated in recombinantly expressed MT proteins by methods of MS spectrometry and spectrophotometry (Palacios et al. 2011). However, it is sometimes difficult to distinguish between “metal affinity” and “metal selectivity” in practice. For example, the metal binding constant of MT complexes with Cd^2+^ is many times higher than with Zn^2+^ (Kägi and Schäffer 1988). A putative metal binding preference of an MT for Cd^2+^ over Zn^2+^ could therefore simply be due to its higher binding affinity for Cd^2+^. In such cases, it is useful to check the presumed MT selectivity for Cd^2+^ also in relation to Cu^+^, which has an even higher affinity for MT thiolate complexes than Cd^2+^ (Krężel and Maret 2017).

The term “metal conformity” was proposed recently (Valsecchi et al. 2023) and refers to the conformational stability of an MT, indicating by how much metal ions are accommodated within the protein in an optimal way that leads to a single, well defined energy minimum in its folding configuration. The method of choice to assess these properties is solution NMR (Beil et al. 2019).

The term “metal specificity”, unlike the previous definitions, refers to the biological function of an MT in favor of or with the help of a certain metal in vivo, either in the organism of origin or a model organism in which the respective MT is expressed, incorporating all available information of a biochemical, structural and biological nature into the assessment wherever possible (Dallinger et al. 1997, 2020).

## The multifarious world of gastropod MTs

### The lasting impact of Cd on the shaping of Cd-selective MT isoforms

With an estimated 90,000 species and six different clades (Patellogastropoda, Vetigastropoda, Neomphaliones, Neritimorpha, Caenogastropoda and Heterobranchia) (Uribe et al. 2022), Gastropoda (snails, whelks, slugs and limpets) are one of the most diverse animal classes, whose representatives have successfully adapted to a wide variety of marine, terrestrial and freshwater habitats. Although their MTs are derived from a common ancestral form, the resulting diversity of sequences and structures is probably greater than in any other lineage of the animal kingdom (Dallinger et al. 2020).

**Fig. 1:**
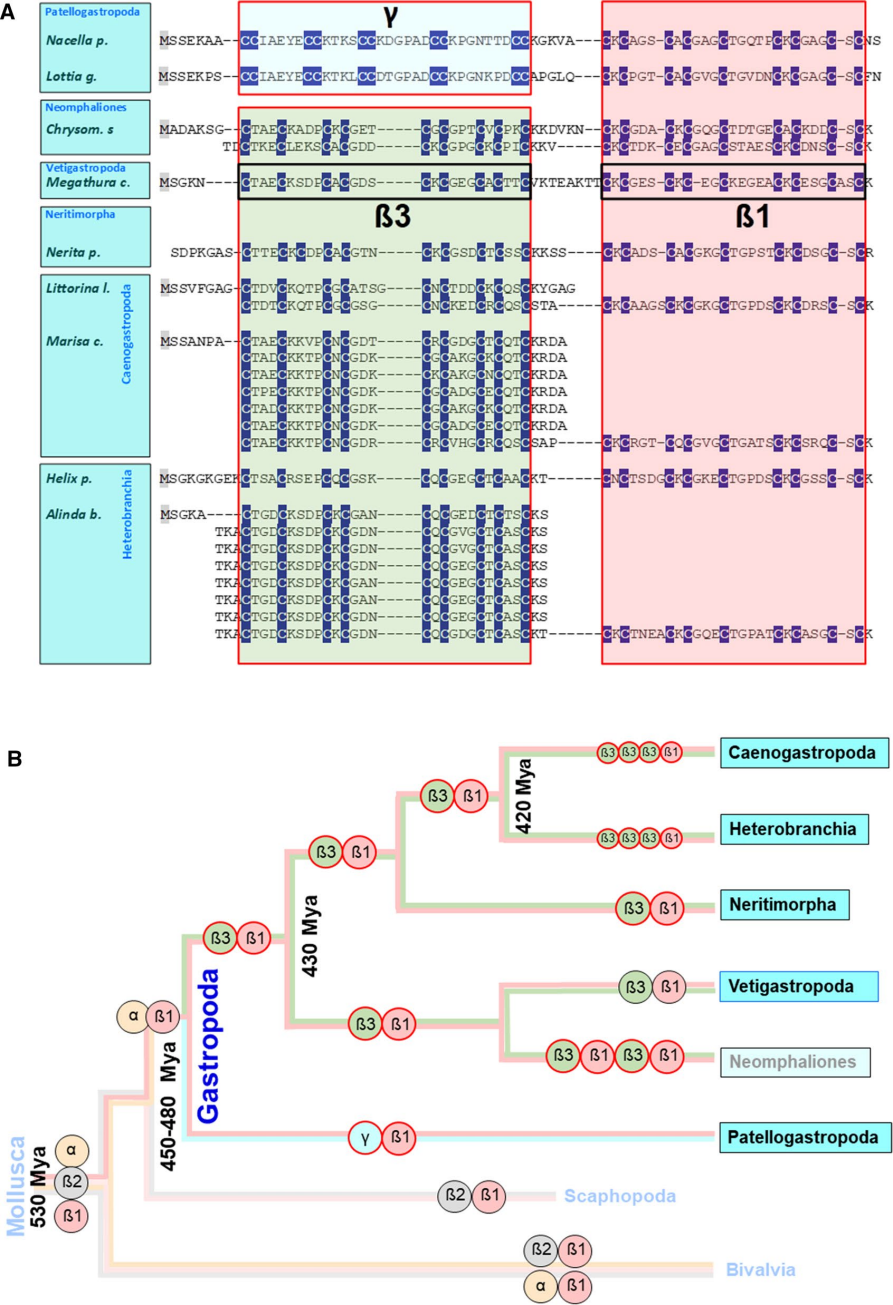
Domain evolution in gastropod MTs. **A** Sequence alignments of MTs and their domains (γ, ß1 and ß3) from selected species of the clades Patellogastropoda (with *Lottia gigantea* and *Nacella polaris*), Neomphaliones (with *Chrysomallon squamiferum*), Vetigastropoda (with *Megathura crenulata*), Neritimorpha (with *Nerita pulligera*), Caenogastropoda (with *Littorina littorea* and *Marisa cornuarietis*), and Heterobranchia (with *Helix pomatia* and *Alinda biplicata*) are shown. Cys residues (C) are highlighted in white letters and underlaid in dark blue. Cd-selective domains are shown in red-framed, non-selective domains in black-framed boxes. Multiple domains are aligned one below the other. **B** Topology of evolutionary branches of Mollusca and Gastropoda with different domains symbolized by colored circles framed in black (unknown or lacking metal selectivity) or red (for Cd-selectivity), and with Greek letters for the different domains). The presence of multi-domain-MTs in Caenogastropoda and Heterobranchia is symbolized by three concatenated β3 domains. The age of some branching points is given very approximately due to contradicting indications in the literature, as derived from (Jörger et al. 2010; Zapata et al. 2014; Wanninger and Wollesen 2019)

It’s a fact that within the MTs of the main gastropod groups, from the beginning of their evolution in the Ordovician (~ 450 million years ago) to the species living today, there has been a continuous development towards optimization of an innate, structurally determined Cd selectivity (Palacios et al. 2017; Beil et al. 2019; García-Risco et al. 2023) (Fig. 1). This selectivity goes hand in hand with a modular structure of the gastropod MTs consisting of at least two different protein domains, the origin of which was apparently already laid out in the earliest precambrian representatives of the phylum Mollusca, of which the class of gastropods is the most successful branch (Calatayud et al. 2021c). In most gastropod MTs, an N-terminal ß3 domain is linked to a C-terminal ß1 domain (Gil-Moreno et al. 2015) (Fig. 1A). Each of these short-chained peptides is about 3.5 kDa in size and contains a tetrahedrally arranged metal-thiolate cluster with nine Cys residues and three Cd^2+^ ions each (Dallinger et al. 2001a, b; Palacios et al. 2011; Beil et al. 2019). As shown in Fig. 1B, the Vetigastropoda are so far the only gastropod lineage for which evidence of Cd-selective MTs is lacking (Pérez-Rafael et al. 2012). However, as only one MT within the Vetigastropoda has been characterized in detail for its metal-binding properties, this finding should be considered preliminary. Moreover, according to the latest, as yet unpublished findings, Cd-selective MTs have indeed been discovered in the group of Neomphaliones, whose phylogenetic position as a sub-clade of Vetigastropoda or an independent lineage next to them is still unclear (Uribe et al. 2022). Figure 1 also shows that MT evolution in gastropods led to the invention of a new N-terminal domain (ß3), which has become established in all gastropod lineages except limpets (Patellogastropoda) (Dallinger et al. 2020). In this latter group, which originated about 450–430 Myr ago as a split from the other gastropod orders, the otherwise usual ß3 domain was replaced by a new domain called γ (García-Risco et al. 2023). In addition, several snail species from the Neomphaliones, Caenogastropoda and Heterobranchia groups have evolved so-called multidomain MTs. They always consist of a conserved C-terminal ß1 domain directly linked to two or more N-terminal concatenated ß3 domains (as in Caenogastropoda and Heterobranchia), or of alternating ß3 and ß1 domains (as in Neomphaliones) (Fig. 1A). This has led to a multiplication of the binding stoichiometry for Cd^2+^ (or other divalent metal ions) in the corresponding MTs (Pedrini-Martha et al. 2020). The capacity to form multidomain MTs seems to be an ancient character of molluscs (Jenny et al. 2004, 2016; Nam and Kim 2017; Calatayud et al. 2022) and has also been observed in the class of bivalves and in other animal phyla, too (Calatayud et al. 2018).

The innate structural Cd selectivity of gastropod MTs has been experimentally documented so far in several species of snails, slugs and limpets from almost all major gastropod clades, mainly using two different analytical approaches: electrospray ionization mass spectrometry (ESI–MS) of MT metal complexes for mass determination and, in some cases, nuclear magnetic resonance spectroscopy (NMR) for 3D-structure elucidation of MT Cd^2+^ and Zn^2+^ complexes in solution. Both approaches were complemented, where necessary and/or possible, by metal titration experiments using additional spectroscopic methods such as circular dichroism (CD) spectroscopy and inductively coupled plasma atomic emission spectroscopy (ICP-AES). In all cases, MT metal complexes for analysis were obtained by recombinant expression in metal-exposed *E. coli* cultures (Palacios et al. 2011; Beil et al. 2019). Remarkably, both approaches independently confirmed the Cd-selective character of MTs from the same species (Palacios et al. 2017; Baumann et al. 2017).

Although the strength of metal complexation within the MT domains is determined by the ranked metal-specific binding affinity of the different metal ions to the thiolate Sulphur atoms (Kägi and Kojima 1987), the actual binding selectivity depends on important additional factors, such as structure, side chain bulkiness and charge of non-coordinating amino acid residues in the vicinity of the Cys metal binding sites (Pérez-Rafael et al. 2014; Palacios et al. 2014; García-Risco et al. 2020; Pedrini-Martha et al. 2020). The combination of these structural features may have ultimately led to the loss, attenuation or enhancement of the Cd-selective properties of gastropod MTs (Palacios et al. 2011).

The Cd selectivity of snail MTs undoubtedly plays an essential physiological role in the detoxification of this metal released into the environment by anthropogenic pollution. Hence, Cd selectivity of snail MTs in this context confers on them a Cd-specific function (Dallinger and Berger 1993; Dallinger et al. 1997; Höckner et al. 2011a, b). In fact, most marine, freshwater and terrestrial snails have developed some Cd tolerance thanks to their MT system which, as a rule with some exemptions, accumulates and detoxifies the metal in the animals' midgut gland (Dallinger 1993; Bebianno and Langston 1998; García-Risco et al. 2023; De Silva et al. 2023). When the detoxification capacity of the Cd-specific MTs in the snail tissues is exhausted, the toxic effects of the metal become apparent and lead to a series of pathological alterations such as programmed cell death, disruption of cellular structures, oxidative stress, and others (Chabicovsky et al. 2004; Hödl et al. 2010; De Silva et al. 2023; Gnatyshyna et al. 2023).

However, Cd must have already played an essential role in the shaping of Cd-selective MTs in geological timescales during gastropod evolution, independent of recent anthropogenic metal immissions into natural realms (Dallinger et al. 2020). Indeed, numerous geological, palaeontological and geochemical publications have documented the geogenic, mostly volcanic, emission of high concentrations of Cd (Porȩbska and Sawłowicz 1997; Xia et al. 2008; Quezada-Hinojosa et al. 2009; Liu et al. 2013; Liu et al. 2017) and other toxic metals during the Earth's latency period, to fossil evidence of their toxic effects as evidenced by anatomical malformations (Vandenbroucke et al. 2015). If so, then it could be assumed that Cd selectivity was an ancestral feature of MTs in general, and must have been more widespread in earlier geological periods than is now apparent in modern MT variants (Calatayud et al. 2021a, 2021b).

### Exploring pathways to Cd selectivity in basal snail clades: the play with ancestral and novel MT domains

**Fig. 2:**
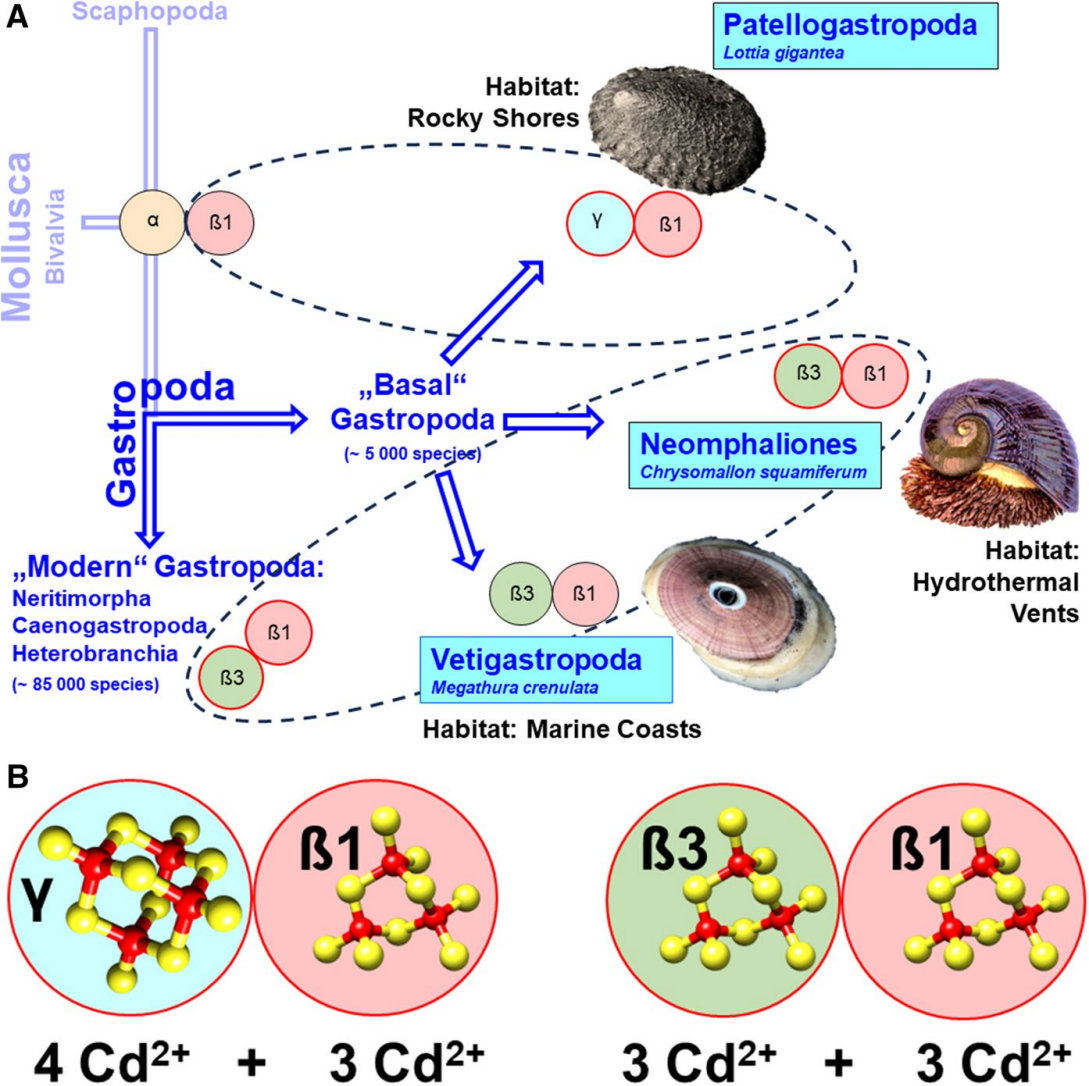
**A** Evolution of Cd-selective MTs and their domains “basal” and “modern” Gastropoda. **A** “Basal” Gastropoda (sometimes also referred to as “Primitive” Gastropoda) (Salvini-Plawen and Haszprunar 1987), shown as derived from Mollusca and with their sister clade Scaphopoda (in light blue) comprise the clades of Patellogastropoda, Vetigastropoda and Neomphaliones (which may perhaps be included into the Vetigastropoda) (Uribe et al. 2022), shown in blue boxes. “Modern” Gastropoda (Frýda et al. 2008) consist of the clades of Caenogastropoda, Heterobranchia and their sister clade Neritimorpha. For single species (*Lottia gigantea*, *Chrysomallon squamiferum*, *Megathura crenulata*), their typical habitats are indicated (in black letters). Different domains are symbolized by colored circles framed in black (unknown or lacking metal selectivity) or red (for Cd selectivity), and with Greek letters for the different domains. **B** The Cd-selective MTs of *Lottia gigantea* (left side) and of *Chrysomallon squamiferum* (right side), symbolized by colored and framed circles (as in **A**), and with numbers of Cd stochiometry for each of the participating domains

A major split in the evolutionary history of Gastropoda occurred at the transition from the Cambrian to the Ordovician period, about 480 million years ago (Fig. 1B). This split gave rise to the primitive branch of Patellogastropoda (the true limpets), and a second branch comprising all other extant gastropod lineages. About 460 million years ago, this latter branch was split up again, giving rise to the basal Vetigastropoda clade (with the lineage of Neomphaliones probably included into the latter) and the three clades of Neritimopha, Caenogastropoda and Heterobranchia clades, with Neritimorpha considered as sister clade of the latter two (Uribe et al. 2022). These three clades can be regarded as “modern” in many respects (Frýda et al. 2008) and today comprise the majority of extant gastropods, with about 85,000 species. They comprise several lineages which were able to colonize terrestrial and freshwater habitats (Vermeij and Watson-Zink 2022). Contrary to them, the basal Patellogastropoda along with the Vetigastropoda and Neomphaliones (Uribe et al. 2022), are considered as “basal” or "primitive" snails (Salvini-Plawen and Haszprunar 1987), with a conservative estimate of about 5000 extant species (Fig. 2A). It also appears that a number of MT variants in the "basal" Gastropoda group (most of them with Cd selectivity) consist of domains of different lineage (see Fig. 1A for their sequences): In particular, the “basal” Gastropoda share the α and ß1 domains with the class of Bivalvia (i.e. the sister class of Gastropoda) (Calatayud et al. 2021c) (Fig. 2). On the other hand, they posses with γ and ß3 novel MT domains that do not exist in the class Bivalvia (Fig. 2A). In evolutionary terms these are, therefore, so-called apomorphic characters that appear for the first time in the class of gastropods. The changing combinations of MT domains in 'basal' Gastropoda can therefore be interpreted as an evolutionary fingerprint of past attempts to test each domain for Cd selectivity in a process of positive selection. For example, the γ domain, which is highly efficient in terms of Cd selectivity (García-Risco et al. 2023), appears to have evolved as an apomorphic trait in certain families of limpet species such as Lottiidae, Nacellidae and Patellidae (Calatayud et al. 2021c) (Fig. 2A). The efficiency of this domain in terms of its Cd^2+^ binding properties lies not only in its particularly strong Cd selectivity, but also in its increased binding stoichiometry for this metal. This allows it to bind four instead of three Cd^2+^ ions per domain compared to all other snail MT domains, in a likely adamantane cage structure (García-Risco et al. 2023) (Fig. 2B). Apparently, these features are laid down in the domain's primary sequence, with an increased number of Cys residues arranged in multiple Cys-Cys repeats (Fig. 1A). Thus, in combination with the ancient mollusc ß1 domain, the whole MT protein of *Lottia gigantea* and other species from the family of Lottiidae can bind seven instead of six Cd^2+^ ions (Fig. 2B). In contrast to Lottiidae, the MT of the keyhole limpet, *Megathura crenulata* from Vetigastropda (Fig. 2A) contains an non-metal selective MT that consists, as seen in modern Gastropoda, of an ancestral ß1 domain combined with a novel ß3 domain (Lieb 2003; Pérez-Rafael et al. 2012). In contrast to the different domain combinations that have been realized and tested in the "basal" group of gastropods, only one combination (ß1/ß3) prevailed in the "modern" gastropods, where it became the canonical MT of most extant snail species (Figs. 1A, 2A). Notably, the Cd selectivity of MTs in several families of „basal” gastropods may have fostered their adaptation to extreme marine environments such as rocky shores, low-oxygen deep-sea regions and habitats located near hydrothermal vents or in cold hydrocarbon seeps (Geiger and Thacker 2006). Life in such realms is such a challenge that only a tiny proportion of marine organisms have adapted to these harsh conditions (Rampelotto 2013; Sogin et al. 2020). Among them are many endemic species of “primitive” snails and limpets including, for example, *Bathyacmaea lactea* from deep-sea cold methane seeps (Zhang et al. 2016; Liu et al. 2021), and *Chrysomallon squamiferum* (the “Scaly-Foot Snail”) from hot hydrothermal vents of the Indian Ocean (Chen et al. 2015; Sun et al. 2020) (Fig. 2). By accumulating huge amounts of Cd and other metallic trace elements, animals in some of these habitats derive their energy from chemosynthetic processes that do not rely on solar energy for their maintenance (Vrijenhoek 2013). Although extremophilic species are found in all extant gastropod clades, the group of “basal” Gastropoda is relatively over-represented when considering the total number of extant species in “basal” (~ 5000) versus “modern” gastropods (~ 85000) (Waren and Bouchet 1993; Sasaki et al. 2010). To date, the conquest of extremophile sites by gastropods can be traced back to the Middle Triassic (Geiger and Thacker 2006; Vrijenhoek 2013), whereas Cd selectivity in snail MTs must have been present since the Ordovician (Dallinger et al. 2020) (Fig. 1B).

Despite the apparent metal tolerance and Cd accumulation capacity of extremophilic gastropods (Cunha et al. 2008; García-Risco et al. 2023), the evolution of their Cd-selective MTs was probably not driven by the colonization of metal-rich hydrothermal vents, given that these habitats are of relatively recent origin and tend to be instable, vulnerable and ephemeral (Vrijenhoek 2013). Instead, adaptation of snails and limpets to such habitats may have been facilitated by their capacity to express Cd-selective MTs. This ability may also have allowed many species of limpest living in less harsh habitats to tolerate elevated levels of Cd contamination in their environment (Noël-Lambot 1980; Noel-Lambot et al. 1980; Bebianno et al. 2003).

### From sea to land, from saltwater to freshwater: the fate of snail MTs across habitat transitions

The adaptation of marine gastropod lineages to terrestrial or freshwater habitats required a profound anatomical redesign of major organ systems, such as the external respiratory organs, but also a change in physiological functions, like water balance or excretion (Vermeij and Dudley 2000). Transitions may have been facilitated through pre-adaptation of marine gastropod species by colonizing marginal marine realms at the sea-land interface of estuarine and intertidal habitats, littoral splash zones or mangrove environments (Krug et al. 2022). As far as we know today, such transitions have occurred several times independently since the Cretacious (Strong et al. 2007; Vermeij and Watson-Zink 2022), but only in gastropod lineages from the "modern" Neritimorpha, Caenogastropoda and Heterobranchia clades (see above and Fig. 2). A preliminary estimate of the number of species that have successfully adapted to life on land or in freshwater puts the number at 4000 freshwater and 24,000 terrestrial species (Strong et al. 2007).

**Fig. 3:**
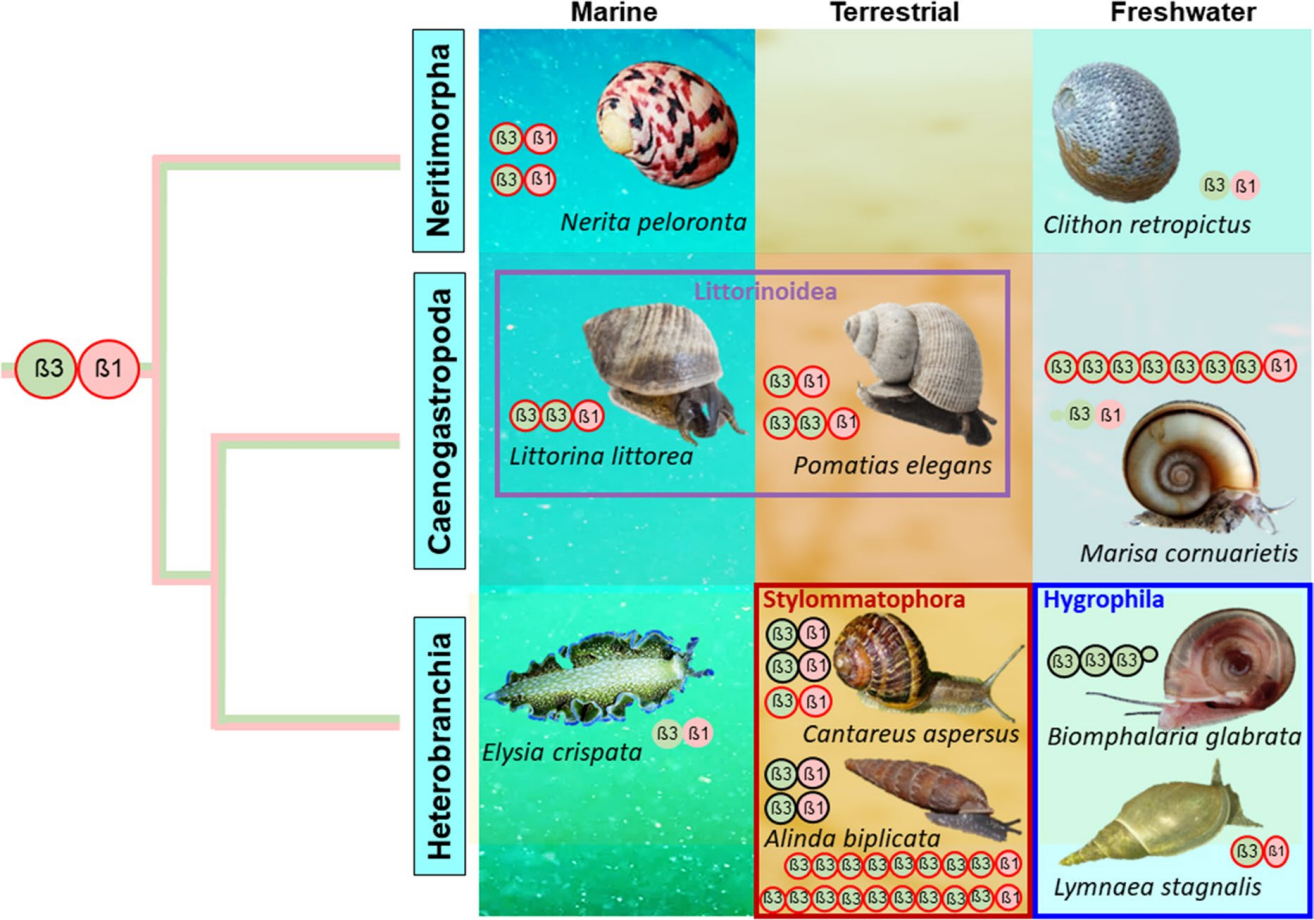
Branching topology of the “modern” Gastropod clades Neritimorpha, Caneogastropoda and Heterobranchia, represented by pictures of selected species (in black italic letters) from either marine, terrestrial and freshwater habitats, with habitat transitions within same clades (in boxes across habitat borders) or habitat adaptations of complete lineages (in boxes within habitat borders), shown with their domains and domain combinations, with each domain symbolized by colored circles with black (unknown or non-selective binding properties) or red frames (for Cd selectivity) and with indication of domain identity by black Greek letters

The conquest of new habitats is also accompanied by changes in the concentrations and availability of trace elements and metals in the new environment, requiring special adaptations of the metal metabolism of the snail species concerned. As mentioned in an earlier publication, the background Cd concentration of the substrates (water, soil, vegetation, etc.) on or from which the snails live can increase 10 to 100-fold at the boundary between marine and terrestrial habitats. In contrast, uncontaminated freshwater habitats usually have 100 to 1000 times lower Cd concentrations than soils (Dallinger et al. 2020). One might assume that the uptake of metals through the gastrointestinal tract is more important in terrestrial snails than in aquatic species, where gill or skin absorption is usually predominant. However, there are several publications showing that terrestrial snails can also absorb metals through the skin and freshwater snails through the gastrointestinal tract (Cœurdassier et al. 2002; Croteau et al. 2007). Overall, therefore, the diet and lifestyle of snail species appear to be a more important factor in metal uptake, regardless of the habitat in which they live (Wang and Ke 2002). Besides a digestive sorting organ (the stomach), all snails have a midgut gland (also called hepatopancreas or digestive gland) as their main absorption and metabolic organ (Salvini-Plawen 1988; Haszprunar 2009), producing digestive enzymes and absorbing nutrients. Minerals and carbohydrates are also stored in this organ, and especially metallic trace elements are sequestered and detoxified in certain midgut gland cells (Dallinger and Wieser 1984; Dallinger 1993; Brooks and White 1995; Włostowski et al. 2014; El Mageed et al. 2023). Interestingly, however, the cellular pathways and biochemical fates of the three metals Zn, Cu and Cd in this organ show remarkable metal-specific differences, with different ratios of their involvement in the composition and metabolism of MT isoforms (Dallinger et al. 1997; Bebianno et al. 2003; Höckner et al. 2011a, b; Dvorak et al. 2019a, 2019b). The midgut gland itself is connected with the alimentary tract via paired ducts, through which nutrients, minerals and metals are transported (Hödl et al. 2010). In so far all gastropod species, the midgut gland is also the main organ of MT expression (Bebianno et al. 1992; Chabicovsky et al. 2003; Benito et al. 2017; Dvorak et al. 2018; Gnatyshyna et al. 2023). In spite of the many habitat transitions in the group of “modern” Gastropoda, the primary structure and the basic compositional plan of their MT domains has remained astonishingly unvaried (Fig. 3). What changes, however, is the number and relative position of the two modular domains ß3 and ß1 within the MT protein and the number of MT isoforms in a lineage- and habitat-specific manner. In both, Caenogastropoda and Heterobranchia, multi-domain MTs have evolved independently, with typically two or more N-terminal ß3 domains linked to a C-terminal ß1 domain. Preliminary data suggest that even long-chained multi-domain MTs form concatenated string-like molecules instead of globular or clustered aggregates (unpublished data) (Pedrini-Martha et al. 2020). A crucial indication of the evolutionary independence of multidomain MTs is the observation that, despite homology in the basic domain arrangement at the protein level, the gene structures of md-MTs in species from different lineages can be fundamentally different. For example, the MT gene of *Biomphalaria glabrata* from the order Hygrophila has a typical exon–intron structure, while the two multidomain MT genes of *Alinda biplicata* from the closely related order Stylommatophora have no introns at all (Pedrini-Martha et al. 2020). Recent unpublished results suggest, moreover, that such fundamental differences in gene structure also exist between the multi-domain MTs of species from the Caenogastropoda and Heterobranchia clades. For many MTs of “modern” Gastropoda, an innate Cd selectivity has been documented, including both, two-domain and multi-domain MTs. This holds for the three-domain MT and its allelic variant (LitliMT and LitliMTvar2) of the marine perwinkle, *Littorina littorea* (Palacios et al. 2017), and the three-domain MT isoform (PeMT1) of its closely related, but terrestrial snail, *Pomatias elegans* (Littorinoidea) (Schmielau et al. 2019); the multi-domain MT isoform of the freshwater Caneogastropod species, *Marisa cornuarietis* (unpublished data); the two-domain CdMTs of terrestrial helicid species like *Arianta arbustorum* (Palacios et al. 2011), as well as the two multi-domain MT isoforms (A.b.9md-MT and A.b.10md-MT) of the closely related Clausiliid species *Alinda biplicata* (Stylommatophora) (Pedrini-Martha et al. 2020) (see Fig. 3). Interestingly, however, there are also some exceptions from this rule. For example, one of the two MT isoforms (NpeMT1) of the marine snail *Nerita peloronta* (Neritimorpha clade) exhibits a clear binding preference for Zn^2+^ over Cd^2+^, while the second one (NpeMT2) shows a higher binding preference for Cd^2+^ (García-Risco et al. 2021). It is so far not known, however, whether the two isoforms share Zn and Cd-specific functionalities in vivo.

Yet, most Cd-selective gastropod MTs are involved in Cd-specific functions, primarily in favor of metal detoxification. An exception of this rule is observed in freshwater-adapted snail lineages (freshwater-adapted Caenogastropoda and Hygrophila, see Fig. 3), where the Cd-specific function of metallothioneins (even when Cd-selective) appears to be reduced and weakened. In all other gastropod lineages, however, the Cd-specific function of their Cd-selective MTs is largely confirmed by a bundle of related data at the molecular, biochemical, cellular, and physiological levels. For example, the genes of these Cd-selective MTs show a pronounced constitutive and Cd-specific inducibility of their expression upon exposure to this metal, whether under field or laboratory conditions (Höckner et al. 2011a; Baurand et al. 2016; Benito et al. 2017; Dvorak et al. 2018; Schmielau et al. 2019; Pedrini-Martha et al. 2020; Pedrini-Martha et al. 2021). In contrast, exposure to other metals or stress factors does not result in a similarly high MT induction (Palacios et al. 2011; Pedrini-Martha et al. 2016). The increased mRNA level is reflected by a respective increase of the Cd-selective MTs at the protein level, as shown by protein fractionation studies using chromatographic methods (Langston and Zhou 1987; Dallinger and Berger 1993; Bebianno and Langston 1998; Dallinger et al. 2004c; Hispard et al. 2008; Dvorak et al. 2018) or MT quantification assays (Berger et al. 1995; Dallinger et al. 2004a, 2004b). Upregulation of gastropod CdMT genes occurs predominantly in the midgut gland, where they are expressed in absorptive, digestive and calcium cells (Chabicovsky et al. 2003; Benito et al. 2017; Dvorak et al. 2018). As a result, abnormally high Cd concentrations can accumulate in the animals' midgut gland (Dallinger and Wieser 1984; Hispard et al. 2008; Schmielau et al. 2019; Pedrini-Martha et al. 2020). At the same time, Cd-exposed snails show an astonishing tolerance to Cd exposure in terms of their survival (Baurand et al. 2015; Włostowski et al. 2014; Hödl et al. 2010), which only breaks down at very high metal concentrations (Chabicovsky et al. 2004; Manzl et al. 2004). Similar results in many other species of Caenogastropoda and Heterobranchia suggest that the metal-specific functionality of their Cd-selective MTs is a phylogenetic feature that first appeared in the marine ancestors and is particularly conserved in species that have adapted to terrestrial conditions (Dallinger et al. 1989a, b; Itziou and Dimitriadis 2011; Schmielau et al. 2019).

**Fig. 4:**
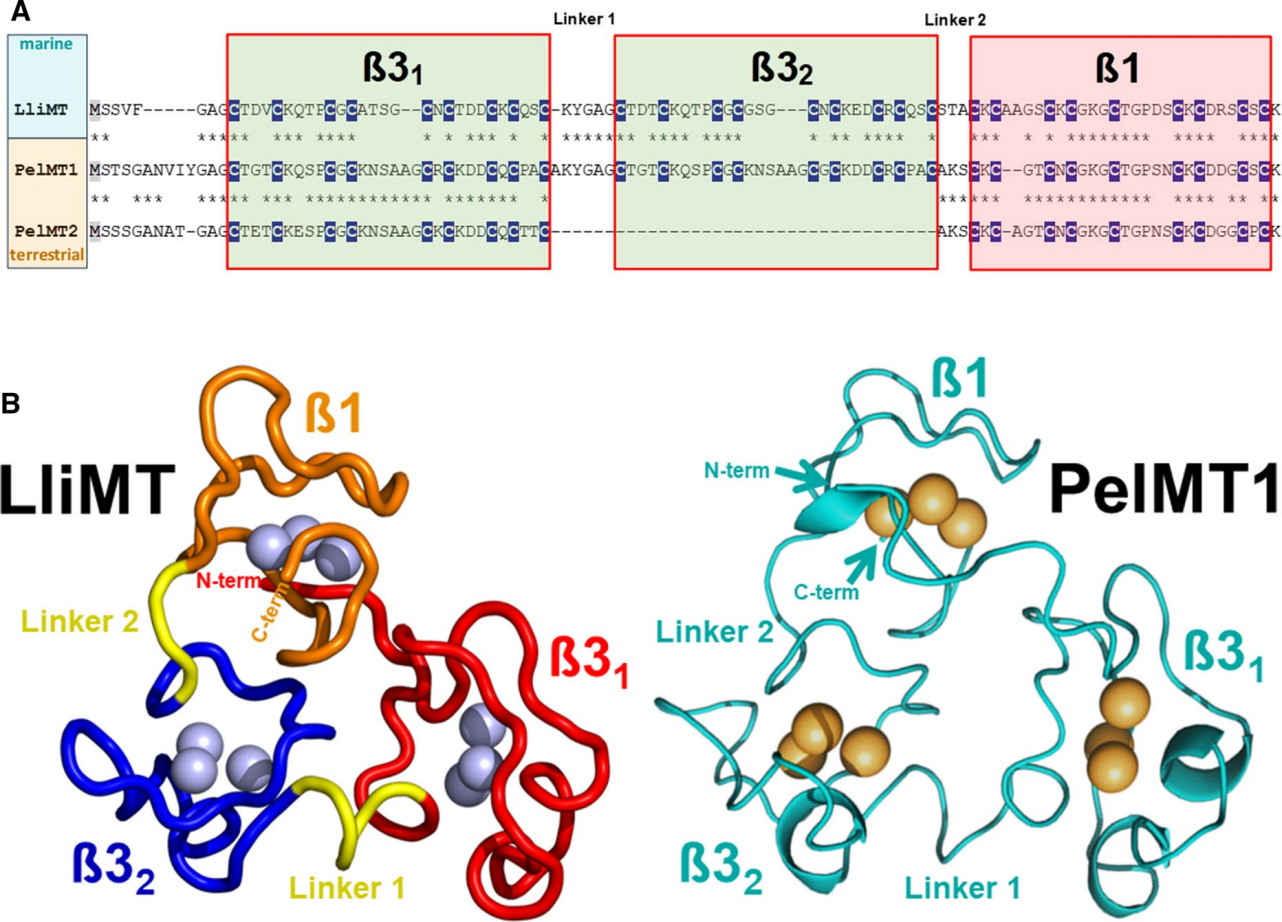
MT sequences and 3D structure of three-domain-MTs form the closely related caenogastropod species adapted to marine habitats (*Littorina littorea*) and to terrestrial conditions (*Pomatias elegans*). **A** Sequence alignments of Cd-selective MTs from *Littorina littorea* (LliMT) and *Pomatias elegans* three-domain (PelMT1) and two domain MTs (PelMT2). Cys residues (C) are highlighted in white letters and underlaid in dark blue. All domains are Cd-selective and shown in red-framed boxes. Linker sequences between domains are also indicated. **B** NMR-derived 3D structure of LliMT rom *Littorina littorea* (left side) with domain backbones in different colours (orange for ß1 and red and blue for ß3_1_ and ß3_2_, respectively), with interdomain linker stretches in yellow. Cd^2+^ ions within the metal binding clusters are represented by blue spheres. On the right side in light blue color the hypothetical 3D structure of PelMT1, derived from 3D modelling calculations with the structure of LiMT as a template (Schmielau et al. 2019), with the three domains ß1, ß3_1_ and ß3_2_. Cd^2+^ ions within the metal binding clusters are represented by yellow spheres

This is particularly evident in the Cd-selective MTs of the marine periwinkle *Littorina littorea* and the terrestrial winkle *Pomatias elegans*. Both species belong to the family of Littorinidae within the clade Caenogastropoda. Ancestral species of this family, such as *Littorina littorea*, live in the transitional marine intertidal zone, while *Pomatias elegans*, as a descendant of marine periwinkles, has adapted to terrestrial life (Fig. 3). Nevertheless, the primary structures of the CdMTs from the two species are surprisingly homologous to each other, with the only difference that *Pomatias elegans* possesses two isoforms, one of which with three metal binding domains (two N-terminal ß3 and one C-terminal ß1 domains), the other one with two domains as many other gastropod MTs (Schmielau et al. 2019) (Fig. 4A). The recombinant wild-type LliMT from *Littorina littorea* and two truncated variants were investigated for metal selectivity towards Cd^2+^ and Zn^2+^ by CD spectrometry and ESI-TOF–MS (Palacios et al. 2017). It was found that both, the wild type and the truncated variants of this species, showed significant selectivity towards both metal ions. Nevertheless, the metal complexes of all three peptides showed a stronger structuring with Cd^2+^ than with Zn^2+^ (Palacios et al. 2017), clearly indicating their binding preference for Cd^2+^ over Zn^2+^. Remarkably, this finding was later confirmed, among others, by ^15^N relaxation data upon elucidation of the 3D structure of LliMT using solution NMR (Baumann et al. 2017) (Fig. 4B). It should be noted here that this study was the first to succeed in completely elucidating the 3D structure of a mollusc MT, and thus, for the first time, also the structure of a multi-domain MT (Baumann et al. 2017). The Cd selectivity of this protein and the rapid up-regulation of its gene upon Cd exposure suggest that its Cd-specific function is dedicated to detoxifying this metal, with an important contribution from digestive gland cells (Vega et al. 1989; Zaldibar et al. 2007; Benito et al. 2017). The Cd detoxification function may also be important under environmental stress conditions such as anoxia or freezing, when energy reserves are limited and the intracellular availability of metals may be subject to rapid changes (English and Storey 2003). A strong Cd accumulation and Cd-specific upregulation of the respective MT genes (PelMT1 and PelMT2) was also observed in the midgut gland of the terrestrial snail *Pomatias elegans*, a close relative of *Littorina littorea* (Schmielau et al. 2019). Due to the high similarity of the primary structure of these two peptides (LliMT and PelMT1) (Fig. 4A), and with the available 3D structure of the LiMT as a template (Baumann et al. 2017), an attempt was made to design the 3D structure of PelMT1 using tertiary structure modelling tools. Hence, the proposed 3D structure of PelMT1 appeared to be amazingly similar to the template 3D structure of LliMT (Schmielau et al. 2019). (Fig. 4B). Thus, Littorinidae snails seem to have conserved their Cd-selective detoxification potential during the transition from sea to land by relying on ancestral protein structures.

**Fig. 5:**
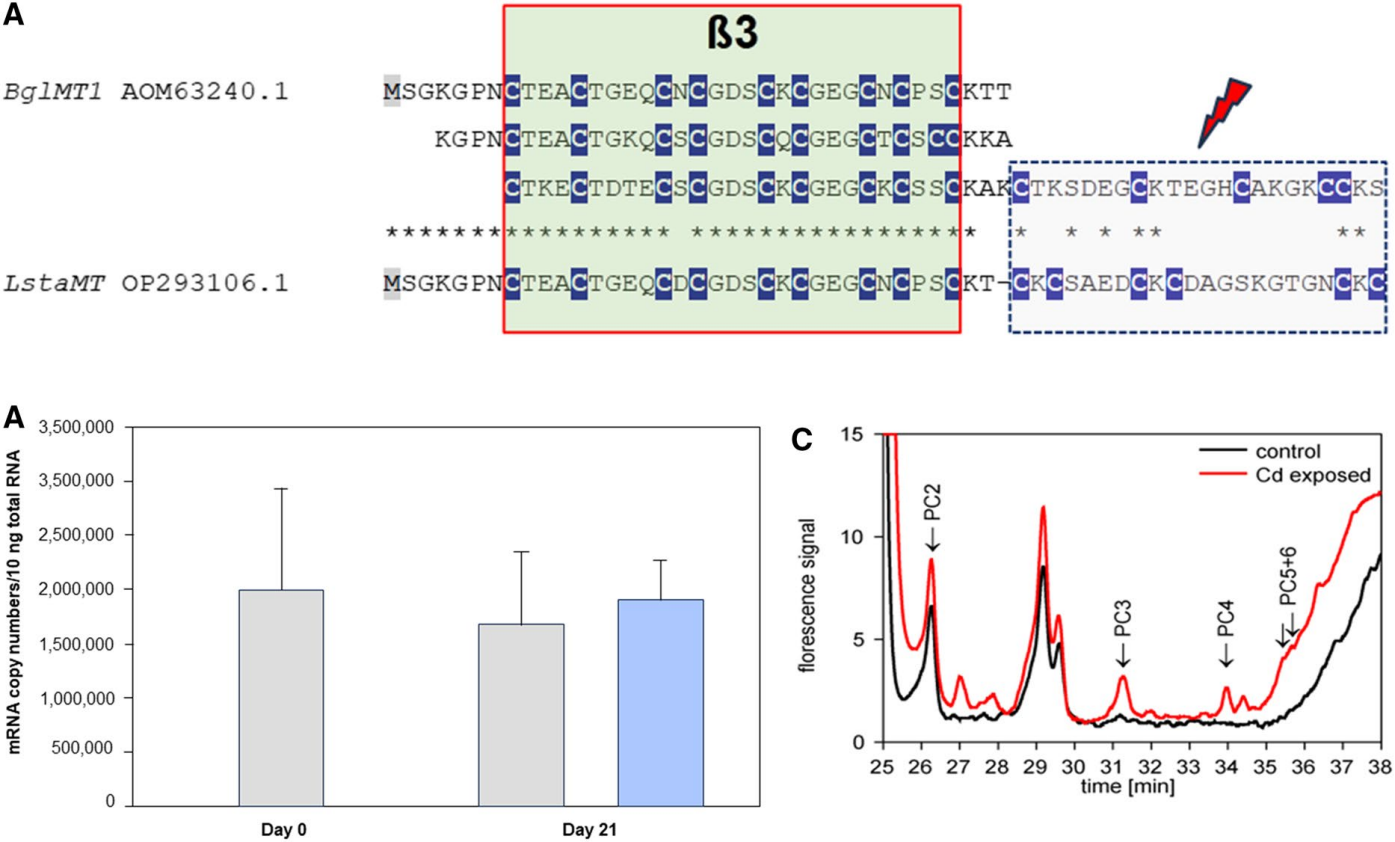
MT sequences, MT gene upregulation and phytochelatin synthesis of freshwater snails from the superorder of Hygrophila (Heterobranchia). **A** Alignment of MT sequences from the freshwater snails *Biomphalaria glabrata* (BglMT1) and *Lymnaea* stagnalis (LstaMT), showing their composition by one or several N-terminal ß3 domains and a C-terminal truncated overhang with a reduced number of Cys residues. Cys positions (C) are highlighted in white letters and underlaid in dark blue. Truncated C-terminal domains are indicated by a red flash arrow. **B** Lacking upregulation of the MT gene (BglMT) of *Biomphalaria glabrata* after Cd exposure through 21 days, compared to controls.** C** Emerging phytochelatin peaks in Cd-exposed *Biomphalaria glabrata* snails (red line) upon chromatography after derivatization and fluorescence detection of thiol groups with monobromobimane (mBrB) (Kawakami et al. 2006) (Dvorak et al. 2019a)

The situation is different in some lineages of Caenogastropoda and Heterobranchia, which have adapted to life under freshwater conditions (Fig. 3). In several species of these lineages, MTs have lost their Cd-selective binding properties and, in many cases, their functionality in Cd-specific detoxification, as demonstrated for the species *Pomacea bridgesii* (García-Risco et al. 2020), or in *Marisa cornuarietis* (publication in preparation). Both species are members of the family Ampullariidae. A deficient induction capacity of MTs under Cd exposure may also be common in other families of Caenogastropoda, as suggested by studies on the freshwater snail *Melanopsis dufouri*, which belongs to the family Melanopsidae (Ureña et al. 2010). The same is true of the Hygrophila, a large suborder of Heterobranchia with actually about 485 extant species (Saadi et al. 2020), which have adapted to life in freshwater habitats. The species *Biomphalaria glabrata*, for example, expresses a deviating MT protein consisting of three concatenated ß3 domains linked to a short C-terminal amino acid overhang (Fig. 5A), lacking any kind of metal selectivity at all (Niederwanger et al. 2017a). Moreover, Cd exposure in this snail does not lead to an upregulation of the respective MT gene, in spite of strong Cd accumulation (Niederwanger et al. 2017b) (Fig. 5B). A similar, weak or lacking response of the MT gene to Cd exposure was also detected in the Hygrophila freshwater snails *Lymnaea stagnalis* (Reátegui-Zirena et al. 2017; Gnatyshyna et al. 2023) and *Physa acuta* (Martínez-Paz et al. 2017).

Instead, these species seem to rely on the compensatory complexation of Cd^2+^ and other metal ions through phytochelatins These are low-molecular weight, Cys-rich peptides synthesized from glutathione by the activity of the enzyme phytochelatin synthase. They were originally discovered in plants (Cobbett 2001), but have more recently also been known from invertebrate animals (Clemens et al. 2001; Vatamaniuk et al. 2001; Liebeke et al. 2013; Bundy and Kille 2014). Their affinity to Cd^2+^ lies, depending on their chain length, in the nano- to femtomolar range, similar to that of CdMTs (Wątły et al. 2021). Thus in the freshwater snail *Biomphalaria glabrata*, Cd exposure leads, instead of MT upregulation, to the synthesis of phytochelatins (Dvorak et al. 2019a), which seem to provide protection against an increasing load of toxic Cd^2+^ (Fig. 5C). Not surprisingly, a similar pattern was observed in *Lymnaea stagnalis*, which is able to compensate its deficient MT system by synthesis of Cd-inactivating phytochelatins (Gonçalves et al. 2016). A poor Cd detoxification capacity was also detected in the related freshwater snail *Physa acuta* (Martínez-Paz et al. 2017). An evolutionary rationale for the loss of Cd-specific functionality in these MTs can only be proposed with some reservations, as it is not yet clear how widespread this deficiency is in freshwater snails. However, it has been argued that evolutionary transition of snails from marine or terrestrial to freshwater habitats was accompanied by significant reductions in background Cd concentrations and metal availability (Dallinger et al. 2020). This could certainly be a possible explanation for the weakening or lacking Cd-detoxifying capacity of MTs in many freshwater snails.

### Recent diversification of Cd and Cu selectivity and metal-specific functionality in MT isoforms of helicid snails

**Fig. 6:**
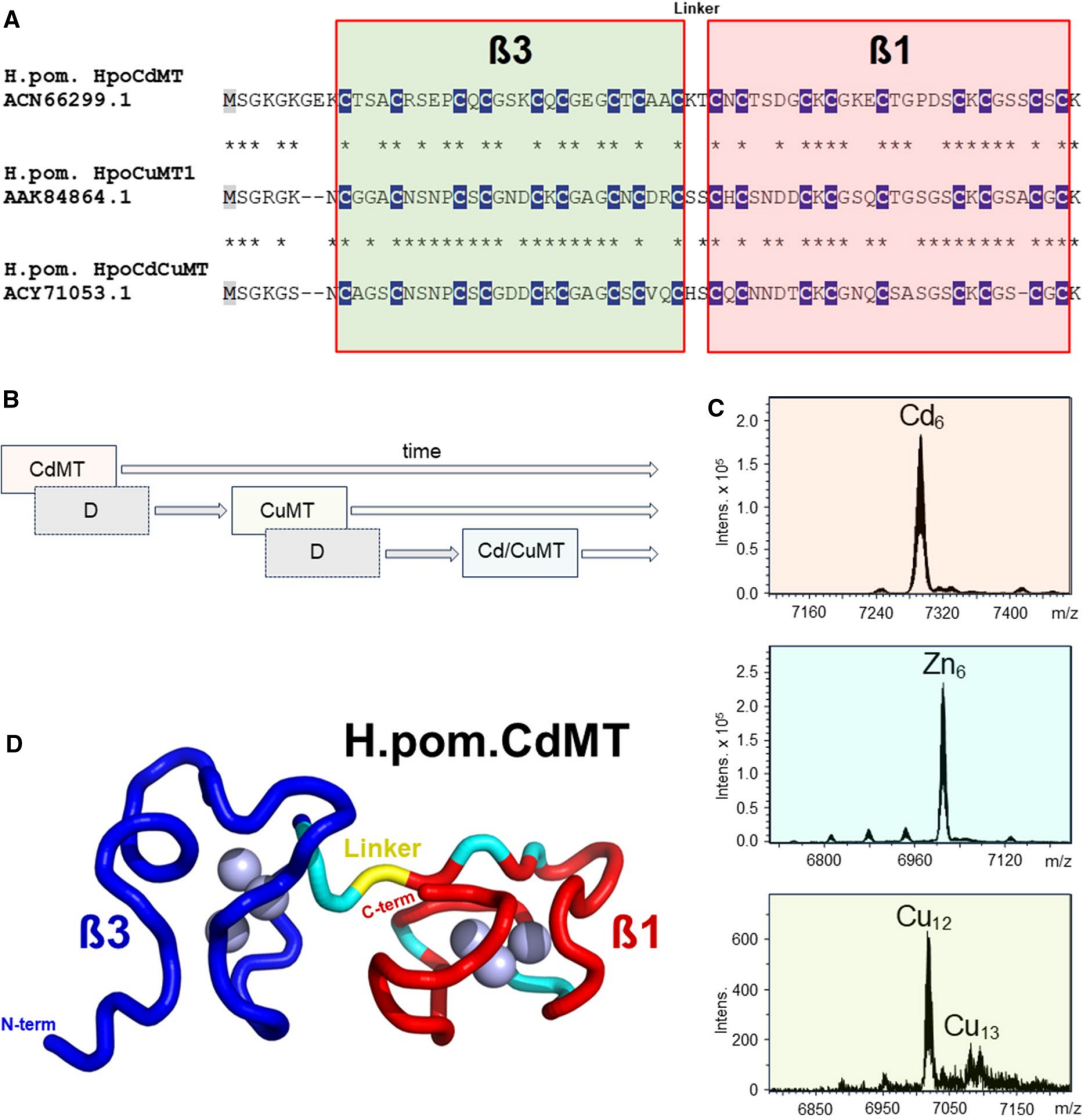
MT sequences of helicid MT isoforms, gene evolution, metal selectivity signals, and 3D structure of the MT gene from the Roman snail, *Helix pomatia*. **A** Alignment of sequences of the Cd-selective (HpoCdMT), the Cu-selective (HpoCuMT1), and the non-selective MT isoform (HpoCdCuMT) from the terrestrial Roman snail, *Helix pomatia*. Cys positions (C) are highlighted in white letters and underlaid in dark blue. The interdomain linker sequence is also indicated. **B** MT isoform evolution in helicid snails by repeated gene duplications of an ancestral Cd-selective MT (CdMT), giving rise to a Cu-selective MT (CuMT) and, eventually, to a non metal-selective MT (Cd/CuMT). **C** ESI–MS signals of the Cd-selective MT, the Zn-loaded CdMT, and the Cu-selective MT from *Helix pomatia*, modified after (Palacios et al. 2011). **D** NMR-based 3D structure of the Cd-selective MT isoform of *Helix pomatia* (H.pom.CdMT), showing the protein backbone with the N-terminal ß3 (in blue) and the C-terminal ß1 domains (in red). Also shown are amino acid stretches (in cyan) contributing to conformational exchange effects in the Zn-loaded variant of H.pom.CdMT (Beil et al. 2019). The interdomain linker is indicated in yellow. Cd^2+^ ions in the metal clusters are shown as blue spheres

One of the major snail lineages that have successfully adapted to terrestriality are Stylommatophora from the clade of Heterobranchia, an order including pulmonate land snails and slugs, with about more than 20,000 to 35,000 extant species (Razkin et al. 2015; Krug et al. 2022). Stylommatophora include well known species such as the edible Roman snail (*Helix pomatia*), the French Escargot (*Cantareus aspersus*), or well-known fruit and garden pests such as the slug *Arion vulgaris*. Preliminary chronological estimates place the origin of Stylommatopohora radiation at around 112 Mya (Jörger et al. 2010), and the onset of the Helicidae radiation at around 38 Mya (Razkin et al. 2015). It is therefore a young and thus "recent" lineage in the evolutionary history of snails (Dallinger et al. 2020). It was a great surprise to discover that snails of the Helicidae family, such as the Roman snail (*Helix pomatia*), express two metal-selective MT isoforms: A Cd-selective variant with a clear function related to Cd detoxification; and a Cu-selective variant that is apparently responsible for the homeostatic regulation of Cu (Dallinger et al. 1997). Subsequently, it was found that both Cd- and Cu-selective MT isoforms are present as two-domain MTs with the canonical ß3 and ß1 domains in all Helicidae species studied so far, including *Cantareus aspersus*, *Cepaea hortensis*, *Cepaea nomoralis*, and *Arianta arbustorum* (Palacios et al. 2011; Dallinger et al. 2020). Finally, a third MT isoform was discovered in these helicides, but it has no metal selectivity and is often expressed only in a very low concentration range (Höckner et al. 2011a, b; Pérez-Rafael et al. 2014) So far, however, its function remains unknown, playing probably only a minor role in the metal balance of adult snails (Höckner et al. 2011b). The primary structures of all three isoforms are surprisingly similar, and the Cys positions are conserved in all three peptide chains (Fig. 6A). The differences in their metal-selective binding properties must therefore be mainly due to the amino acid residues between the Cys positions (Pérez-Rafael et al. 2014), as already mentioned and explained above (see chapter “The lasting impact of Cd on the shaping of Cd-selective MT isoforms”). As suggested by phylogenetic inference (Dallinger et al. 2020), the three isoforms have evolved through successive duplication events of an ancestral gene encoding a Cd-selective MT, with the non-metal selective Cd/CuMT isoform eventually emerging in the last step of these duplication events (Fig. 6B). Particular attention was focused on the two metal-selective MT isoforms of helicid snails. Their metal selectivity was first studied by ESI–MS methods in recombinantly produced CdMTs from *Helix pomatia*, showing that each of the two metal-selective isoforms yield best folded, homogeneous complexes of distinct structure and stoichiometry only with its cognate metal ion, i.e. CdMT with Cd^2+^ and CuMT with Cu^+^ (Palacios et al. 2011, 2014; Gil-Moreno et al. 2015). In contrast, when recombinant MTs are forced to associate with non-cognate metal ions (e.g. CdMT with Zn^2+^ or Cu^+^ and CuMT with Cd^2+^ or Zn^2+^), minimum energy conformations are not reached and metal complexes of variable stoichiometries are formed (Palacios et al. 2014). According to these studies, the stoichiometry of the metal-selective snail MTs is 6 Cd^2+^ ions per protein molecule for CdMT and 12 Cu^+^ ions per protein molecule for CuMT (Fig. 6C). A convincing correspondence for these values was found in CdMT and CuMT preparations isolated in vivo from native snail tissues (Gehrig et al. 2000). In line with the high chemical similarity between Cd^2+^ and Zn^2+^, most snail Cd-selective MTs also show a high propensity to form well-structured complexes with Zn^2+^, thus supporting the possible existence of functional divalent metal ion-selective MTs such as those suggested in the urochordate, *Oikopleura dioica* (Calatayud et al. 2021a). A recent study has shown, moreover, that in spite of their strong similarity, Cd^2+^ and Zn^2+^ bind in distinctly different ways and with different binding preferences to the respective ß and α domains of the mammalian MT-2 (Peris-Díaz et al. 2021). This could also apply to the snails' Cd-selective MTs, whose Zn^2+^ complexes differ markedly from the better folded corresponding Cd^2+^ complexes, which could have implications for their chemical classification and biological functions (García-Risco et al. 2021). The evolutionary optimization of the binding preference of Cd-selective snail MTs for Cd^2+^ over Zn^2+^ has independently been confirmed by solution MMR, using the 3D structure of the CdMT of the Roman snail (*Helix pomatia*) as a model (Beil et al. 2019) (Fig. 6D). Indeed, the C-terminal ß1 domain of the snail CdMT shows amino acid stretches (in cyan) in the peptide chain that contribute to conformational exchange effects in the Zn^2+^-loaded but not in the Cd^2+^-loaded form of the CdMT, suggesting that the CdMT structure is stiffer and more solid when loaded with the cognate metal ion. This indicates that the ß1 domain is critical for the process of Cd^2+^ loading and retention and suggests that the *Helix pomatia* CdMT isoform is evolutionarily optimized for Cd^2+^ binding (Beil et al. 2019). The dominant role of the C-terminal ß1 domain in the dynamics of metal loading was also confirmed by NMR for the three-domain CdMT of *Littorina Littorea* (Baumann et al. 2017). This would also explain the fact that the primary structure of ß1-domains is usually better conserved across snail CdMTs compared to the corresponding β3-domains (Dallinger et al. 2020).

In *Helix pomatia*, the biological function of this isoform as a Cd-detoxifying protein is supported by its structure and function at the gene level. Interestingly, the Roman snail was found to possess one of the largest animal MT genes known (Egg et al. 2009). In its promoter region, the gene contains four Metal-Responsive Elements (MREs) that can be upregulated by a mollusc-specific MTF-1 (publication in preparation). Interestingly, the gene also contains intronic repeats of putative transcription factor binding sites (TFBs), which have been implicated in transcriptional regulation and environmental stress response (Egg et al. 2009). Consistent with the proposed Cd-specific detoxifying function of the encoded MT, the gene may be up-regulated several-fold in response to Cd exposure, whereas it is little or not affected by other metals and environmental or physiological stressors (Palacios et al. 2011). In contrast, the corresponding CuMT gene of *Helix pomatia* is surprisingly unresponsive and does mostly not appear to be highly upregulated by exposure to Cd or other metals (Dallinger et al. 2004a; Palacios et al. 2011).

### Toxic cadmium, hidden zinc and dosed copper: the cellular counterpart

**Fig. 7:**
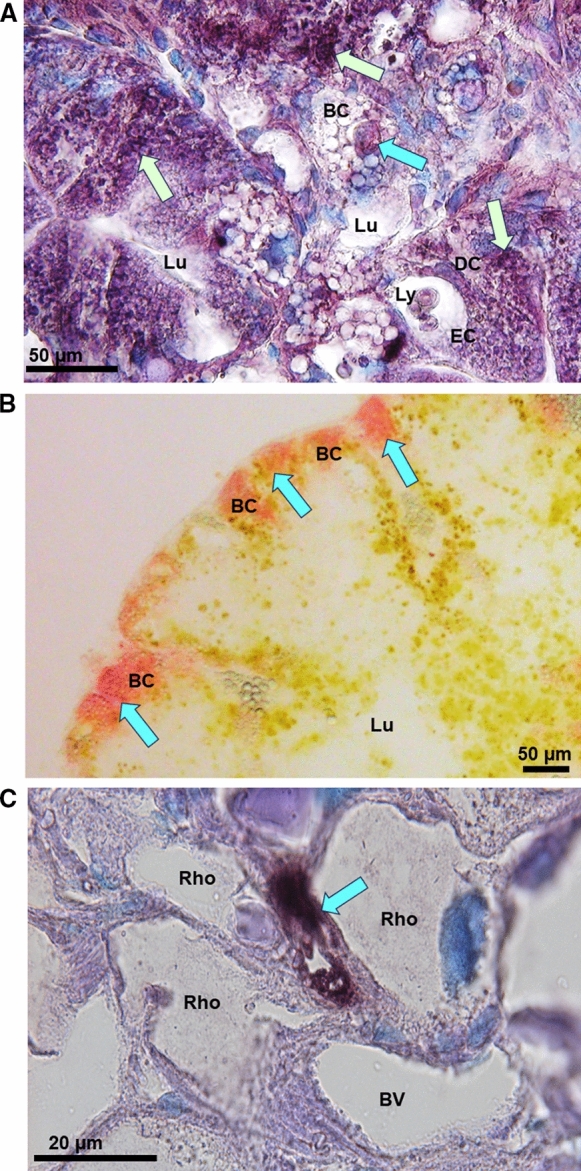
Light microphotographs of midgut gland tissue sections of *Helix pomatia*. **A** Midgut gland cells of tubular epithelia showing the Lumen (Lu) surrounded by Basophilic Cells (BC), Digestive Cells (DC) and Excretory Cells (EC) with CdMT mRNA expression signals (dark violet spots) indicated by light green arrows, and a condensed cell body in programmed cell death within a basophilic cell (light blue arrow), **B** Midgut gland tubulus with central Lumen (Lu) and peripheral Basophilic Cells (BC) with Zn visualized by Dithizone reactions (blue arrows and red color). **C** Midgut gland section showing a group of Rhogocytes (Rho) delimiting a Blood Vessel (BV), with CuMT mRNA expression signals (blue arrow, dark violet spot)

Cd is a cytotoxic agent (L’Azou et al. 2014; Lee and Thévenod 2020; Branca et al. 2020). Its detoxification in the snail’s tissues occurs mainly in the midgut gland and goes hand in hand with strong metal accumulation in the midgut gland cells. In gastropods, the midgut gland consists mainly of two to three cell types, called digestive cells, basophilic cells or calcium cells, and excretory or thin cells (Triebskorn and Köhler 1996; Marigómez et al. 1998; Dallinger et al. 2001a; Lopes et al. 2001; Chabicovsky et al. 2004; Mohammadein et al. 2013; Mleiki et al. 2018; El Mageed et al. 2023). These cells form a monolayer epithelium which delimits the finely branched tubules that run through the midgut gland and open into the alimentary tract via the paired midgut gland ducts (Lobo-da-Cunha 2019). Apart from epithelial tubular cells, the midgut gland tissue of gastropods also contains connective tissue cells and so-called rhogocytes, which play an important role in Cu accumulation and regulation (Mason et al. 1984; Dallinger et al. 2005). Despite expression of Cd-selective MTs and their involvement in metal detoxification, snails remain susceptible to elevated Cd concentrations. When exposed to the metal, initial effects are seen immediately after intoxication, with rapid histological and morphological alterations in digestive gland cells (Vega et al. 1989; Hödl et al. 2010), even before the onset of MT gene upregulation (Benito et al. 2017). Eventually, Cd stress leads to an increased tissue turnover with cell replacements and changes of cell type compositions in the digestive gland (Vega et al. 1989; Zaldibar et al. 2007; Hödl et al. 2010). This provides an important line of defence against the effects of this metal, but also against other environmental stressors that require rapid adaptation to changing environmental conditions.(Zaldibar et al. 2008; Mohammadein et al. 2013). It is a reversible response mechanism, as long as the stressing factor is not too intensive (Zaldibar et al. 2007). During this process, excess amounts of toxic Cd^2+^ are inactivated by MT and sequestered in the lysosomal system upon MT deficiency, overload, or degradation (Bebianno and Langston 1998; Chabicovsky et al. 2004; Desouky 2006; Cunha et al. 2008; Mleiki et al. 2018). At the same time, however, metals and other types of cellular stressors lead to leakage of the lysosomal system, so that toxic ions and metabolites can be released back into the cytoplasm, where they induce pathological responses, including, among others, programmed cell death and oxidative stress (Chabicovsky et al. 2004; Hödl et al. 2010; Gnatyshyna et al. 2023) (Fig. 7A). Additional environmental stressors such as desiccation, freezing or oxygen deprivation can exacerbate these dynamic processes, making the presence of effective MTs even more necessary (English and Storey 2003; Pedrini-Martha et al. 2016). Thus, snail Cd-specific MTs play an ambivalent role: they mainly protect their hosts, but can also trigger cytotoxic processes. An important difference with vertebrate MTs is that the Cd-selective helicid MTs are very good at binding Zn^2+^ ions in vitro, but under in vivo conditions they contain only trace amounts of this metal, if any (Dallinger et al. 1997, 2020). This also applies to snails or slugs that had previously experimentally been exposed to Zn^2+^, as shown by purified MT preparations from their native tissues (Dallinger and Berger 1993; Dvorak et al. 2019b). In spite of high uptake rates of Zn in the snail midgut gland, the metal was predominantly found in pellet fractions after high-speed centrifugation, and in low molecular weight compounds significantly lower than MT fractions upon chromatography (Dallinger et al. 1989a, b, 1993). This suggests that in snails, the biochemical and cellular pathways of the essential trace element Zn^2+^ are strictly separated from those of the toxic Cd^2+^. This is consistent with the idea that the evolution of Cd-selective MT in snails was primarily or solely dedicated to the function of detoxifying this metal without affecting the metabolism of chemically similar and essential zinc (Dallinger et al. 2020). It cannot be excluded that at very low cellular Cd^2+^ concentrations, these MTs may also play a certain role in Zn^2+^ metabolism. However, it is more likely that Zn^2+^ homeostasis in snail tissues is related to the expression and activity of non-metal-selective MT isoforms, which have been discovered in many snail species in addition to the metal-selective isoforms (Höckner et al. 2011b; Pérez-Rafael et al. 2012). Recently, it has also been shown that in some terrestrial gastropods, Zn^2+^ is apparently bound far from the MT pool to at least one distinct low-molecular-weight ligand with a mass of 837 Da and a calculated isotopic formula of C_12_H_20_N_3_O_6_Zn^+^. This would indicate the presence of a nicotianamine-Zn^2+^ complex (Dvorak et al. 2019b) and would be a new finding in the animal kingdom. Nicotianamine has previously been recognized as major chelator and regulator of Zn^2+^ metabolism in plants (Clemens et al. 2013), where it may act, with its very high stability (Kd = 1.6 10^–11^ M at pH 7.4), as a regulator of Zn homeostasis (Krężel and Maret 2016). In the midgut gland cells of gastropods, this low-molecular weight Zn^2+^ pool is clearly separated from the MT proteins, and is localized exclusively in basophilic cells, which also contain highly mobile Ca reserves in the form of calcium granules (Dvorak et al. 2019b) (Fig. 7B).

No less surprising was the detection of a Cu-selective MT isoform in helicid snails (Dallinger et al. 1997). In contrast to the Cd-selective MT, this isoform exhibits a strict cell-specific expression pattern, being localized exclusively in the so-called rhogocytes of snails (Chabicovsky et al. 2003) (Palacios et al. 2011). These are particular cells, sometimes also called pore cells, found within Mollusca (Haszprunar 1996), and long known for their involvement in metal regulation (Simkiss and Mason 1983). In Roman snails, rhogocytes are found scattered in all tissues, with a particularly high abundance in the mantle and midgut gland, where they can be visualized by *in-situ*-hybridization of their respective mRNA (Dallinger et al. 2005) (Fig. 7C). Rhogocytes are apparently involved in snail Cu regulation and are also important expression sites of hemocyanin, the gastropods Cu-containing respiratory protein (Kokkinopoulou et al. 2014). This has given rise to the hypothesis that in the snail rhogocytes, CuMT may serve as a Cu^+^ donator to nascent hemocyanin molecules (Dallinger et al. 2005), as has previously been suggested for the CuMTs in crustaceans, which also possess hemocyanins (Brouwer et al. 1986). Indeed, recent studies have shown that genes of both, *CuMT* and a hemocyanin isoform (*CaH aD*), can be upregulated by Cu exposure in the midgut gland of the helicid garden snail, *Cornu aspoersum* (also called *Cantareus aspersus*) (Pedrini-Martha et al. 2021). So far, however, the Cu donator hypothesis through CuMT could not be verified. It should also be noted that in the evolutionary history of gastropods, hemocyanins are as old or even older than MTs (Lieb and Markl 2004; Markl 2013), without the emergence of Cu-selective MTs in most other gastropod clades (Dallinger et al. 2020). It is now thought that the Cu-selective MTs in snail rhogocytes are more involved in the homeostatic regulation of physiological Cu^+^ activities within these cells, allowing a dosed transfer of Cu^+^ to nascent haemocyanin without being involved in this transfer themselves. In any case, the physiology of the snail organism seems to distinguish very precisely between the pathways of the three metals cadmium, zinc and copper.

## Conclusions: what can we learn from the evolutionary history of a family of metal-handling proteins?

Studying the evolutionary history of a protein family in combination with experimental approaches and analytical methods is helpful in many ways. From the present example of MT evolution in gastropods, the following conclusions and highlights can be derived.

1. Gastropoda, with approximately 90,000 existing species and a great diversity of habitat adaptations and lifestyles, offer enormous potential for studying MTs and their variation in both genetically closely and very distantly related species. This will allow tracing a time course of MT evolution in different lineages, leading to a classification of MT sequences and their metal binding properties in terms of ancestral, derived and modern properties. It also illuminates the evolutionary potential of the MTs superfamily as a hole, contributing to our understanding of how MT structures could diversify and which properties they could assume, not seen in MTs of other taxa.
2. In particular, these studies demonstrate that in Gastropoda, MTs have evolved from ancestral domain combinations whose metal-binding properties are not yet clear, mainly because respective studies are lacking. However, the predominantly Cd-selective ß1 domain found in all gastropods was already present in molluscan ancestors and is the only MT domain that has survived into modern gastropods. This suggests that Cd-selective MTs were already present at the base of gastropod radiation and that this selectivity was gradually optimized in different lineages. Interestingly, it was shown that Cd selectivity may also have been predominant in tunicates, a basal clade of chordata (Calatayud et al. 2018).
3. MT evolution in gastropods began with the convergent invention of novel, predominantly Cd-selective MT domains (γ and ß3) in basal clades, whereas other metal selectivities or even losses of selectivity reflect more recent evolutionary events. This may shed more light on other MT families such as vertebrate MTs, too, where metal-selectivity today is the exception rather than the rule. In fact, non-selective MTs appear to be more versatile and diverse in their responses, as they can simultaneously serve different metal-specific needs of the cells (Krężel and Maret 2017; Coyle et al. 2002). Seen in this light, "losing" metal selectivity could have been an evolutionary gain rather than a deficit, implying the emergence of multifunctionality. This makes MT research harder and our understanding of different MT functions more complicated. This is also true of gastropods, where MTs have partially or entirely lost their metal selectivity in some recently emerged clades, without our understanding why these proteins were still retained and evolved. By no means does this mean, however, that MTs are elusive molecules.
4. The work on gastropod MTs has also shown how useful it is to combine bio-inorganic analytical methods with experimental biological approaches in order to better understand their function in vivo. To this end, the biology of metals (such as Cd^2+^, Zn^2+^ and Cu^2+^/Cu^+^) must be taken into account, including their distinct and metal-specific pathways and effects through organs and cells. After all, it is the cell-specific metal need and the cell itself that determine the functional context of an MT, be it metal-selective or not (Palacios et al. 2011; Foster and Robinson 2011).
5. Finally, it can be seen from the example of gastropods that MT research would benefit from greater consideration of evolutionary and environmental aspects. This would also require a more open attitude towards softening the boundaries between bioinorganic chemistry, as well as life and environmental sciences (Jeffrey 2003).
